# IL-15 Signaling Pathways in the Kidney: Established Mechanisms and Hypotheses for Exercise-Associated Stress

**DOI:** 10.3390/biom16101536

**Published:** 2026-10-21

**Authors:** Marina Skorkina, Alexandr Bobylev, Daria Belova, Yan Bravyy, Kerim Mutig, Svetlana Lebedeva

**Affiliations:** 1 Scientific Center of Genetics and Life Sciences, Sirius University of Science and Technology, Sirius 354340, Russia; skorkina.my@talantiuspeh.ru (M.S.); bobylyov.ag@talantiuspeh.ru (A.B.); belova.da@talantiuspeh.ru (D.B.); brave.yr@talantiuspeh.ru (Y.B.); mutig.k@talantiuspeh.ru (K.M.); 2 Institute of Theoretical and Experimental Biophysics, Russian Academy of Sciences, Pushchino 142290, Russia; 3 Department of Pharmacology, Sechenov First Moscow State Medical University, Moscow 119991, Russia

**Keywords:** interleukin-15, skeletal muscle, kidney, podocytes, tubular epithelial cells (TECs), physical activity, acute kidney injury (AKI)

## Abstract

Interleukin-15 (IL-15) is an immunoregulatory cytokine also recognized as a myokine, with experimentally demonstrated cytoprotective and metabolic effects. Renal tubular epithelial cells (TECs) and podocytes express components of the IL-15 receptor system, but the role of IL-15 in exercise-associated renal adaptation remains unclear. This review distinguishes evidence from renal experimental models from hypotheses regarding exercise-induced renoprotection. Cell-culture and preclinical studies support a role for IL-15 in promoting TEC survival and attenuating profibrotic responses. Separate studies in podocytes demonstrate a functional role for STAT5 in autophagy regulation and improvements in mitochondrial fusion and respiration in response to IL-15, with optic atrophy 1 (OPA1) implicated in the latter response. These findings do not establish a single integrated pathway or demonstrate that the same protective mechanisms operate during exercise. Strenuous exercise may transiently increase tubular stress- or injury-associated biomarkers; however, these increases do not necessarily indicate clinically significant acute kidney injury (AKI) and should be interpreted alongside measures of renal function and the clinical context. We outline a conceptual framework that distinguishes potentially protective responses in renal parenchymal cells from IL-15-dependent immune activation that may contribute to injury in selected disease settings. Cytokine availability, exposure duration, mode of presentation, receptor composition, and inflammatory context may influence these outcomes, but their roles in renal responses to exercise remain to be established. An increase in circulating IL-15 alone does not establish its skeletal muscle origin, delivery to the kidney, or biological activity in renal target cells. The key unresolved question is whether IL-15 produced by skeletal muscle in response to exercise reaches renal target cells in a biologically active form and confers measurable renoprotection.

## 1. Introduction

Interleukin-15 (IL-15) is a pleiotropic cytokine with well-established immunological and emerging metabolic functions. Its biological activity depends on its cellular source, availability, mode of presentation, receptor composition, and the state of the responding cells. IL-15 promotes T-cell proliferation, supports the generation of lymphokine-activated killer cells, regulates natural killer (NK) cell function, and contributes to the maintenance and expansion of memory-phenotype CD8^+^ T-cell populations [1,2,3,4]. These immunological functions provide a foundation for investigating its effects in non-immune tissues.

Within the kidney, tubular epithelial cells (TECs) produce IL-15, and primary human TECs have been shown to express the complete heterotrimeric IL-15 receptor complex, comprising IL-15Rα (CD215), IL-2/IL-15Rβ (CD122), and the common γ-chain (γc, CD132), and to respond to IL-15 stimulation [5,6,7,8]. Here, a “functional IL-15 receptor system” denotes the expression of relevant receptor components together with demonstrated responsiveness to IL-15, rather than receptor expression alone. IL-15Rα supports high-affinity cytokine binding and presentation, whereas CD122 and γc mediate intracellular signaling in responding cells [9].

Podocytes also express components of the IL-15 receptor system and respond to exogenous IL-15. Experimental studies have demonstrated IL-15-induced STAT5 phosphorylation, a functional contribution of STAT5 to autophagic regulation and stress resistance, and beneficial effects of IL-15/IL-15Rα treatment in experimental glomerular injury. Separately, IL-15 has been linked to the preservation of mitochondrial fusion and respiratory function in podocytes under diabetic conditions. These findings do not establish identical receptor requirements in TECs and podocytes or a single integrated signaling pathway [7].

IL-15 is also recognized as a myokine expressed by skeletal muscle, and exercise may alter muscle *IL15* expression and circulating IL-15 concentrations [10,11]. However, changes in circulating concentrations alone do not establish skeletal muscle as the cytokine source [12]. In skeletal muscle, IL-15 may exert context-dependent effects that differ between physiological and pathophysiological conditions [13]. It has been proposed to contribute to exercise-associated immune regulation and metabolic flexibility, but these proposed functions should be distinguished from experimentally demonstrated responses. Accordingly, IL-15 should not be assigned a uniformly anti-inflammatory role during exercise or a uniformly pro-inflammatory role under pathological conditions [13].

Experimental studies have reported effects of IL-15 on carbohydrate and lipid metabolism, including modulation of lipogenesis and insulin sensitivity [14,15,16,17]. IL-15 has also been associated with anabolic responses, myogenesis, and attenuation of muscle wasting in selected experimental settings relevant to sarcopenia and cachexia [12,18,19]. Collectively, these findings identify IL-15 as a potential mediator linking muscle biology, metabolism, and immune regulation, although its contribution to physiological exercise responses remains incompletely defined [12,13].

Locally produced renal IL-15 should be distinguished from the putative effects of circulating, muscle-derived IL-15 on the kidney. Experimental evidence supports a role for locally produced IL-15 in regulating TEC survival and inflammatory responses [20]. In renal epithelial models, IL-15 has also been associated with preservation of the epithelial phenotype [6]. Separate preclinical studies of fibrosis have demonstrated attenuation of extracellular matrix accumulation [21]. By contrast, the effects of exercise-associated, muscle-derived IL-15 on renal cells remain hypothetical. Exercise-related changes in muscle *IL15* expression and serum or plasma IL-15 concentrations do not, by themselves, demonstrate secretion from skeletal muscle or delivery to the kidney. Direct evidence that IL-15 produced by skeletal muscle in response to exercise reaches renal target cells at biologically active concentrations is currently lacking. Consequently, renoprotective or adaptive effects attributed to this proposed endocrine signal should be regarded as mechanistic extrapolations rather than established physiological responses.

Evidence for skeletal muscle-kidney communication has emerged from an experimental model of diabetic kidney disease (DKD), in which muscle-derived IL-15 was linked to the preservation of optic atrophy 1 (OPA1)-associated mitochondrial fusion and respiratory function in podocytes and to improved renal outcomes [22]. However, these findings were obtained in a chronic metabolic disease model and cannot be directly extrapolated to transient exercise-associated renal stress.

Exercise has complex effects on kidney physiology. Regular moderate physical activity may support kidney health, whereas strenuous or prolonged exercise can impose hemodynamic, metabolic, and inflammatory stress [23,24]. Transient elevations in tubular stress- or injury-associated biomarkers may occur even when conventional diagnostic criteria for acute kidney injury (AKI) are not met. However, neither biomarker elevation nor rapid normalization alone establishes the presence or absence of clinically consequential injury; interpretation requires measures of renal function and consideration of the clinical context [25]. Exertional rhabdomyolysis [26,27,28], hypovolemia [29], heat stress [30,31], marked alterations in renal perfusion [32], exercise-associated hyponatremia [29], and use of nonsteroidal anti-inflammatory drugs (NSAIDs) [24,33,34] may increase vulnerability to exercise-associated kidney injury. These overlapping factors provide a rationale for investigating mechanisms of renal cellular adaptation and survival during strenuous or prolonged exercise. Despite accumulating evidence regarding the systemic and tissue-specific effects of IL-15, its role in renal responses to exercise remains poorly understood.

Previous reviews have considered IL-15 in exercise physiology [10,35], muscle metabolism [36,37], and kidney disease [38], while related studies have addressed sarcopenia [39] and chronic kidney disease [22,40]. The present review integrates these bodies of evidence while preserving the distinctions between their experimental contexts. It synthesizes findings on IL-15 biogenesis and presentation, skeletal muscle expression, receptor-associated signaling, and functional effects in renal cells, with particular attention to epithelial homeostasis, autophagy, and mitochondrial function.

Three levels of evidence are distinguished: cellular mechanisms demonstrated in the relevant experimental systems; functional effects established in preclinical disease models, including muscle-kidney communication in DKD; and hypotheses concerning renal responses during exercise. This distinction allows the potential relevance of IL-15 to exercise-associated renal stress to be assessed without treating independently demonstrated findings as a validated exercise-induced pathway. The key unresolved question is whether IL-15 produced by skeletal muscle in response to exercise reaches renal target cells in a biologically active form and confers measurable renoprotection.

### Literature Search Strategy and Data Evaluation Principles

To prepare this review, a targeted literature search was conducted in PubMed for articles published up to 15 October 2026. PubMed Central and official publisher websites were used to retrieve full-text articles, and verify bibliographic information. Search strings combined the core terms IL-15, IL-15Rα, and trans-presentation with the following keywords: skeletal muscle, exercise, aging, sarcopenia, kidney, podocyte, tubular epithelial cell, acute kidney injury, diabetic nephropathy, mitochondria, autophagy, muscle–kidney crosstalk, rhabdomyolysis, myoglobin, NF-κB, heat stress, NSAIDs, renal perfusion, kidney injury biomarkers, kidney–muscle axis, renal–muscle crosstalk, and myokines.

Primary experimental and clinical studies were prioritized. Review articles were used to identify relevant research areas and locate primary sources. For key publications, we evaluated the species studied, cell types, cell lines or animal models, participant characteristics, interventions, control conditions, time points, methods, and outcome measures. Sample sizes (*n*) were reported only when they could be unambiguously attributed to the specific finding described.

Data synthesis distinguished among three categories: (1) effects and molecular mechanisms directly demonstrated within the relevant experimental system; (2) mechanistic extrapolations across different cell types, tissues, or experimental conditions; and (3) hypotheses proposed in this review regarding the potential role of IL-15 in physical exercise, aging, and exercise-induced kidney injury. The absence of direct experimental evidence was explicitly indicated throughout the manuscript.

## 2. Molecular Organization and Tissue Distribution of the IL-15/IL-15R System

### 2.1. Molecular Structure of IL-15 and Receptor-Mediated Signaling

Interleukin-15 (IL-15) is a 14–15 kDa glycoprotein expressed by a broad range of immune and non-immune cells [41]. Structurally, IL-15 belongs to the four-helix bundle cytokine family. Its receptor system belongs to the common γ-chain (γc; CD132) cytokine receptor family, which also includes receptors for IL-2, IL-4, IL-7, IL-9, and IL-21 [42,43]. In biological fluids, IL-15 may occur in an uncomplexed form or in association with the high-affinity IL-15 receptor α subunit (IL-15Rα), forming soluble IL-15/IL-15Rα complexes [44,45]. The IL-15 receptor system comprises IL-15Rα, IL-2/IL-15Rβ (CD122), and γc (CD132) [41]. IL-15Rα supports high-affinity cytokine binding, stabilization, intracellular trafficking, and presentation, whereas IL-2/IL-15Rβ and γc mediate intracellular signaling in responding cells [46,47]. IL-15 bound to IL-15Rα can be presented in cis to signaling receptor subunits on the same cell or in trans to those on an adjacent responding cell. In trans-presentation, IL-15 bound to IL-15Rα on a presenting cell engages CD122 and γc on a responding cell; expression of all three receptor subunits on the responding cell is therefore not required. IL-15 can activate several major intracellular signaling pathways, including JAK/STAT [37,48,49], RAS/RAF/MAPK, and phosphatidylinositol 3-kinase/protein kinase B/mechanistic target of rapamycin (PI3K/Akt/mTOR) signaling [50,51,52]. In immune cells, these pathways contribute to survival, proliferation, and metabolic regulation. Their relative involvement depends on cell type, receptor expression, cytokine availability, mode of presentation, and stimulation conditions.

**Figure 1:**
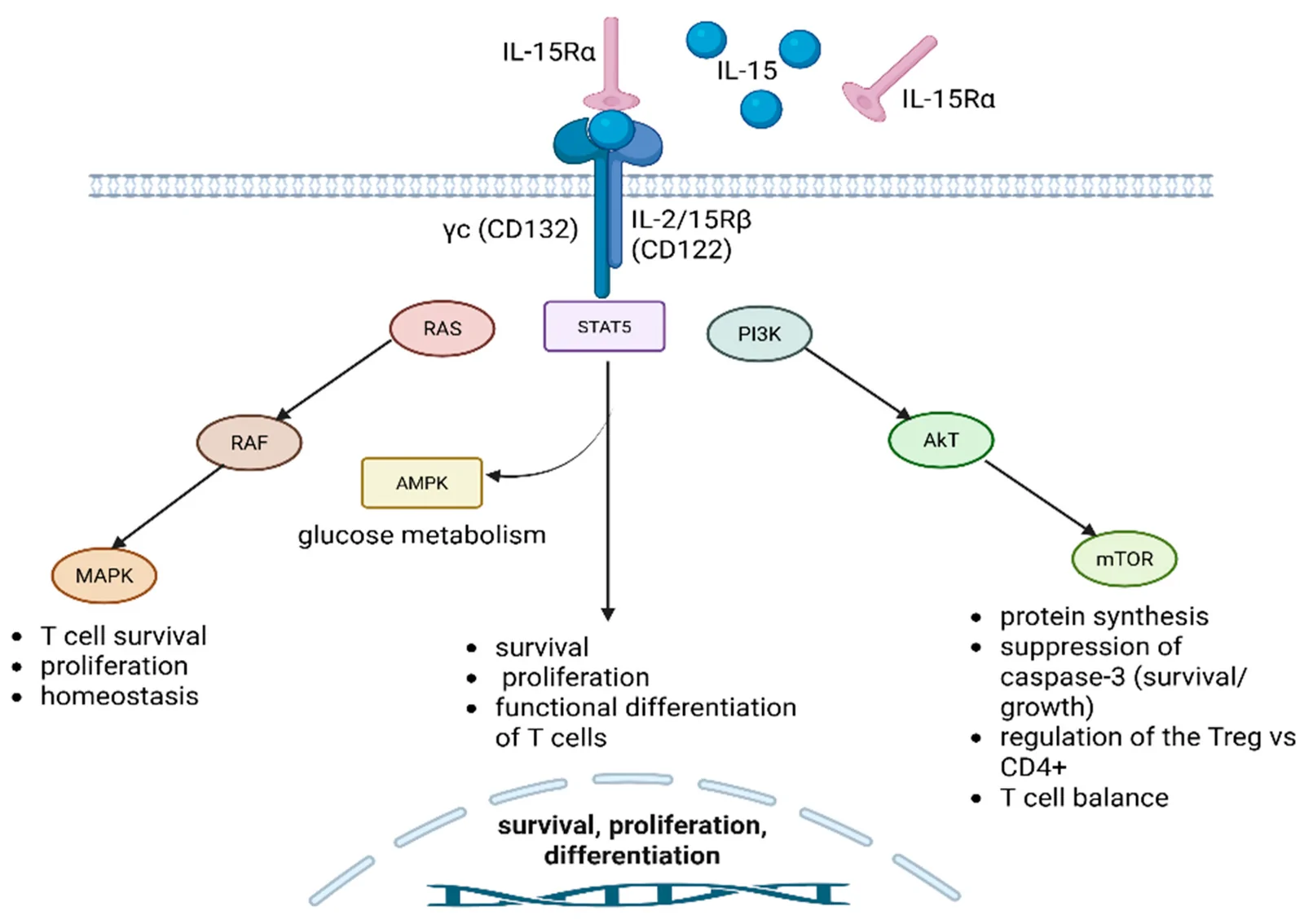
IL-15 receptor organization and major signaling pathways. IL-15 activates three major intracellular signaling pathways: JAK/STAT, RAS/RAF/MAPK, and PI3K/Akt/mTOR. In T cells, JAK/STAT signaling regulates cell survival, proliferation, and functional differentiation and may influence energy metabolism, including glucose metabolism, via interactions with the AMPK pathway. Activation of the PI3K/Akt/mTOR pathway promotes protein synthesis and may inhibit caspase-3 activity, thereby contributing to cell survival and growth, and may regulate the balance between regulatory T cells (Tregs) and other CD4^+^ T-cell subsets. The RAS/RAF/MAPK and PI3K/Akt/mTOR pathways also participate in T-cell survival, proliferation, and homeostatic maintenance. Abbreviations: IL-15, interleukin-15; IL-15Rα, IL-15 receptor α subunit; γc, common γ-chain; STAT, signal transducer and activator of transcription; MAPK, mitogen-activated protein kinase; PI3K, phosphatidylinositol 3-kinase; Akt, protein kinase B; mTOR, mechanistic target of rapamycin; AMPK, AMP-activated protein kinase. Treg, regulatory T cell.

In skeletal muscle cell models, IL-15 has been reported to increase glucose uptake, promote GLUT4 translocation, and influence mitochondrial function. In C2C12 myotubes, IL-15-induced glucose uptake and GLUT4 translocation involved JAK3/STAT3 signaling without a detectable increase in AMP-activated protein kinase (AMPK) phosphorylation [37]. In L6 myotubes, acute IL-15 exposure increased basal glucose uptake and mitochondrial oxidative function in association with AMPK pathway activation [53]. These findings indicate model- and exposure-dependent responses rather than a universal JAK3/STAT3–AMPK cascade. They support effects of IL-15 on muscle glucose handling and oxidative metabolism in experimental systems, but their relevance to endogenous cytokine exposure during physiological exercise remains incompletely defined (Figure 1).

The human *IL15* gene is located on chromosome 4q31 [54,55]. IL-15 expression is regulated at transcriptional, translational, and post-translational levels. Alternative transcript processing generates precursors containing either long or short signal peptides, while the mature cytokine sequence is shared by the two forms [56].

The long signal peptide (LSP)-containing precursor has a 48-amino-acid signal peptide and is associated with entry into the endoplasmic reticulum and subsequent intracellular trafficking. The short signal peptide (SSP)-containing precursor has a 21-amino-acid signal peptide and has been reported to localize predominantly to intracellular compartments, including the cytoplasm and nucleus [55,57]. However, signal peptide identity alone does not determine secretion, and co-expression of IL-15Rα can stabilize SSP-derived IL-15 and facilitate its secretion as a bioactive IL-15/IL-15Rα complex [57,58,59]. Intracellular localization or entry into the secretory pathway therefore does not, by itself, establish the amount of biologically available cytokine released.

IL-15/IL-15Rα complexes can assemble within producing cells and subsequently reach the plasma membrane, where they engage CD122/γc on adjacent cells through trans-presentation. Proteolytic shedding of membrane-associated IL-15Rα can also contribute to the generation of soluble IL-15Rα or soluble IL-15/IL-15Rα complexes. Soluble IL-15/IL-15Rα complexes may remain biologically active and stimulate responding cells without direct contact with the producing cell, although their activity depends on the molecular form and experimental context [60].

These modes of presentation influence the spatial range and temporal dynamics of IL-15 signaling. Experimental comparisons have reported faster, more transient responses to cis-presented IL-15 and slower, more persistent responses to an IL-15/IL-15Rα-based agonist used to model trans-presentation [61]. These findings should not be generalized to all cellular settings, because signaling kinetics also depend on complex stability, receptor trafficking, internalization, and cytokine exposure.

Consequently, measurement of uncomplexed IL-15 alone may not fully capture the availability of IL-15 for receptor-mediated signaling. Distinguishing uncomplexed IL-15 from soluble IL-15/IL-15Rα complexes, together with assessing receptor expression and functional responses in target cells, may provide a more informative characterization of IL-15 availability than circulating concentrations alone. Interpretation also requires knowledge of which molecular forms are detected by the assay [62].

### 2.2. Tissue Distribution and Cellular Organization of the IL-15 Receptor System

*IL15* mRNA has been detected in multiple organs in addition to skeletal muscle, including the placenta [63], kidneys [8], heart [64], brain [65,66,67], bone marrow, thymus, lungs, and liver [68]. Expression of IL-15 and/or IL-15Rα has been reported in epithelial cells [69], fibroblasts [70], keratinocytes [70,71,72], cardiomyocytes [64], macrophages and monocytes [73], astrocytes [66,67,74], and dendritic cells [75]. However, detection of transcripts or receptor components alone does not establish cytokine secretion or functional signaling in a given cell population. IL-15 protein abundance and availability are regulated by translation, intracellular trafficking, protein turnover, and association with IL-15Rα. Accordingly, *IL15* mRNA abundance does not necessarily reflect the amount of secreted or biologically available cytokine.

Within the immune system, IL-15 acts predominantly through trans-presentation [41]. IL-15 complexed with IL-15Rα on the surface of myeloid, stromal, or other presenting cells engages the CD122/γc receptor complex on adjacent responding cells, particularly NK cells and CD8^+^ T lymphocytes [76]. This mode of presentation supports spatially restricted signaling and local target-cell activation without requiring a substantial increase in free circulating IL-15.

Central nervous system (CNS). In the CNS, IL-15 has been implicated in both neuroinflammatory and cell-survival responses [77]. In experimental cerebral ischemia, astrocyte-derived IL-15 has been associated with the recruitment, accumulation, and activation of NK cells and CD8^+^ T lymphocytes [78]. IL-15 blockade also reduced effector responses in NK cells and in both CD8^+^ and CD4^+^ T cells in mice subjected to cerebral ischemia–reperfusion injury [79]. In intracerebral hemorrhage models, IL-15 has been implicated in pathological astrocyte-microglia interactions [80]. Conversely, IL-15 administration reduced neuronal death and modulated stress-associated responses in cultured cortical neurons exposed to experimental ischemia-like conditions [81]. Thus, the net effect of IL-15 in neural tissue appears to depend on the nature and stage of injury, the cellular source of IL-15, the responding cell population, the mode of IL-15 presentation, and the duration and magnitude of signaling.

Cardiovascular system. IL-15 has been detected in inflammatory cells within atherosclerotic plaques [82,83], and circulating concentrations have been associated with atherosclerotic disease [84]. Experimental studies suggest that IL-15 may exert cardioprotective effects during acute myocardial injury. In models of myocardial infarction and myocarditis, IL-15 has been associated with reduced cardiomyocyte death and improved cardiac function [85]. In cardiomyocytes, IL-15-mediated protection against cell death induced by hypoxia or oxidative stress has been linked to STAT3 phosphorylation and PI3K/ERK1/2 signaling [64,86]. These findings indicate context-dependent cardiovascular effects: associations with chronic vascular inflammation coexist with experimentally demonstrated cardiomyocyte protection in selected acute-injury models.

Kidney. In the kidney, TECs both produce IL-15 and express components of its receptor system. In addition to soluble IL-15, a membrane-associated form of IL-15 (mb-IL-15), with an apparent molecular mass of approximately 14–16 kDa, has been described in proximal tubular epithelial cells (PTECs). In normal PTECs, this form is thought to be retained at the cell surface through association with IL-15Rα. Normal PTECs also express a functional heterotrimeric IL-15 receptor complex comprising IL-15Rα, CD122, and γc. These cells exhibit γc-associated JAK3 signaling [6]. Soluble IL-15 has been reported to support PTEC survival and preserve the epithelial phenotype, including maintenance of E-cadherin expression [87].

Immunohistochemical analyses of normal renal tissue have detected IL-2/15Rβ, γc, and JAK3 in TECs in cortical and medullary regions [6]. Together with experimental evidence of endogenous IL-15 production and cellular responsiveness, these observations support the existence of local renal signaling mechanisms. However, tissue localization alone does not establish the mode of cytokine presentation or the relative contributions of autocrine signaling, paracrine signaling and epithelial-immune interactions.

Evidence concerning other nephron segments remains more limited. Receptor components have been detected in distal tubules, identifying these cells as potential IL-15-responsive populations [6]. In the loop of Henle, *IL15RA* expression has been identified in transcriptomic datasets, including single-cell analyses [88]. These observations identify potential sites of IL-15-related activity but do not establish the presence of a complete receptor system or functional responsiveness to IL-15.

Within the glomerulus, podocytes are IL-15-responsive cells. Functional involvement of the IL-2/15Rβ and γc subunits in podocyte responses to IL-15 has been reported [7]. Separate experiments have demonstrated a contribution of STAT5 to autophagy regulation and stress resistance, as well as beneficial effects of IL-15/IL-15Rα treatment in experimental glomerular injury [7].

IL-15 has also been implicated in the regulation of podocyte mitochondrial bioenergetics [22]. Under hyperglycemic conditions, IL-15 increased basal oxygen consumption, ATP-linked respiration, and maximal respiratory capacity, while promoting mitochondrial fusion in association with increased OPA1 expression [22]. These findings suggest that IL-15 may help preserve podocyte mitochondrial function. However, these findings were obtained in experimental models of DKD and do not establish whether a comparable mechanism operates during exercise-associated renal stress.

Overall, the studies reviewed here provide evidence for both local IL-15 production and functional responsiveness in PTEC models, whereas podocyte studies primarily demonstrate responsiveness to exogenous IL-15. For other nephron segments, the evidence is predominantly descriptive and requires further protein-level and functional validation. Representative cellular sources, receptor components, and experimentally reported responses across tissues are summarized in Table 1.

Across organs, the consequences of IL-15 signaling vary with the producing and responding cells, receptor organization, mode of presentation, and injury context. Broad tissue expression should therefore not be interpreted as evidence of equivalent autocrine or paracrine functions in all organs. Against this background, evaluating skeletal muscle as a potential endocrine source of IL-15 for the kidney requires separate assessment of muscle transcript abundance, tissue protein content, and circulating cytokine concentrations, as well as differentiation between acute exercise responses and longer-term training adaptations.

## 3. Skeletal Muscle as a Potential Source of IL-15 for the Kidney

### 3.1. Biological Effects of IL-15 in Skeletal Muscle

Skeletal muscle expresses the *IL15* gene and is considered a potential source of IL-15 [95]. In experimental models, IL-15 has been reported to promote contractile protein accumulation, modulate muscle protein synthesis and degradation, and influence myogenic responses and satellite-cell activity, while attenuating muscle wasting under selected conditions [10,108,109,110]. IL-15 overexpression has also been associated with improved indices of muscle quality and increased autophagy induction, despite reduced muscle mass [111]. However, the responsiveness of myogenic cells to IL-15 does not establish endogenous IL-15 as a universal anabolic factor under physiological conditions [96,97].

Beyond its effects on protein homeostasis, IL-15 may enhance glucose uptake, influence mitochondrial oxidative function, and modulate redox responses [112,113]. Proposed mechanisms involve peroxisome proliferator-activated receptors (PPARs), including PPAR-δ [114], and associated oxidative programs [115]. Separately, JAK3/STAT3 signaling has been implicated in IL-15-induced glucose uptake and GLUT4 translocation in skeletal muscle cells. Nevertheless, the molecular mechanisms linking IL-15 to mitochondrial function and redox homeostasis remain incompletely understood [116].

These metabolic effects vary with cytokine concentration, exposure duration, and cellular context. In L6 myotubes, short-term exposure to IL-15 increased basal glucose uptake and mitochondrial oxidative function [114]. Prolonged exposure produced a biphasic concentration-response relationship, with enhanced mitochondrial oxidative function at lower concentrations and suppression at higher concentrations [117]. These observations describe responses in a specific cell-culture system and should not be generalized to physiological IL-15 exposure during exercise or to other cell populations.

IL-15 has been proposed to contribute to the regulation of the oxidative phenotype of skeletal muscle and fatigue resistance, although the relative contributions of local and systemic signaling remain unresolved. Potential effects on adipose tissue have also been investigated. In cultured adipocytes, IL-15 stimulated adiponectin secretion [118,119]. These findings suggest potential interactions between IL-15 and systemic metabolism, but they do not establish a physiological role for muscle-derived IL-15 during exercise or its protective effects against aging- or disease-associated sarcopenia.

Evidence consistent with endocrine activity comes from mice with muscle-specific IL-15 overexpression. Increased circulating IL-15 was associated with reduced adipose tissue accumulation, whereas elevated intramuscular IL-15 without a corresponding increase in circulating concentrations did not alter body composition. However, constitutive overexpression and exogenous cytokine administration differ from exercise-associated changes in endogenous IL-15 availability. These experimental findings therefore cannot be directly equated with physiological exercise responses [96].

Interpreting these findings requires consideration of the multilevel regulation of IL-15 biogenesis. Muscle *IL15* transcript abundance may vary with muscle fiber-type composition, whereas tissue protein content and biological availability additionally depend on translation, protein turnover, intracellular trafficking, association with IL-15Rα, and release or presentation. Consequently, increased *IL15* mRNA abundance in skeletal muscle does not necessarily indicate higher tissue IL-15 protein levels or enhanced cytokine secretion.

### 3.2. Exercise-Associated Regulation of IL-15 Expression and Circulating Levels

Skeletal muscle can function as an endocrine organ by synthesizing and releasing signaling molecules, including cytokines and peptides collectively termed myokines [120]. These molecules participate in local autocrine and paracrine signaling and may mediate endocrine communication with distant organs, including the brain, adipose tissue, bone, liver, intestine, and pancreas [121,122].

Physical activity may alter *IL15* expression in skeletal muscle and circulating IL-15 concentrations. Responses vary with exercise type, intensity, and duration; training status; participant characteristics; metabolic state; and sampling time [123,124]. Importantly, muscle transcript abundance, intramuscular protein content, and circulating cytokine concentrations may differ in both the magnitude and time course of their responses [125]. For example, 24 h after a single bout of heavy resistance exercise, *IL15* mRNA abundance in the vastus lateralis increased approximately twofold, whereas muscle IL-15 protein content and plasma IL-15 concentrations did not change significantly at any of the investigated time points [95]. In another study, serum IL-15 concentrations increased approximately 5.3-fold immediately after resistance exercise. Muscle *IL15RA* mRNA abundance increased at 4 h, and both *IL15* and *IL15RA* mRNA abundance increased at 24 h [126]. These findings illustrate that changes in circulating IL-15 concentrations and muscle transcript abundance can follow different time courses [126].

Increases in circulating IL-15 have also been reported after endurance exercise [35,117,127]. Significant elevations were observed in untrained young men after 30 min of treadmill running [128] and in athletes after a 35 km trail run with a total elevation gain of 940 m [129].

In a cohort of cancer survivors, circulating IL-15 concentrations increased by 113% immediately after a high-intensity interval exercise (HIIE) bout performed before a 7-month high-intensity interval training (HIIT) program and by 102% after a comparable bout performed at the end of the program [130]. The training program did not significantly alter pre- or post-HIIE IL-15 concentrations. These findings indicate an acute response to individual exercise bouts rather than a demonstrated training-induced increase in resting concentrations.

The exercise protocol may also influence the response. In a study comparing protocols classified by the authors as static and dynamic, resistance exercise increased plasma IL-15 concentrations by approximately 50% in weightlifting athletes, whereas dynamic exercise on a cycle ergometer did not significantly alter IL-15 concentrations [131]. Although this difference suggests that exercise mode may be relevant, it does not isolate the effects of contraction type from other differences between the protocols.

Pooled evidence further emphasizes the distinction between acute responses and adaptations to exercise training. A systematic review and meta-analysis published in 2024 included 15 studies in the acute exercise analysis and 12 in the chronic exercise-training analysis. Circulating IL-15 concentrations increased immediately after acute exercise. The pooled estimate also indicated an increase at 1 h, but this finding was not robust in sensitivity analyses. Substantial between-study heterogeneity limits the generalizability of these results. In contrast, the primary pooled analysis did not detect a statistically significant effect of exercise training on circulating IL-15 concentrations [35]. Taken together, these findings support an average acute exercise-associated increase in circulating IL-15 rather than a uniform response across studies or a consistent increase after training (Table 2).

Individual studies have reported increases, decreases, or no significant changes in circulating IL-15 after exercise [136,140]. Differences in study populations, exercise protocols, sampling schedules, and analytical methods complicate comparisons and highlight the need for standardized assessment.

Interpretation requires a clear distinction between skeletal muscle *IL15* mRNA abundance, intramuscular IL-15 protein content, and serum or plasma IL-15 concentrations. These outcomes represent different biological processes: mRNA abundance reflects the balance between transcript production and degradation; tissue protein content reflects synthesis, turnover, retention, and release; and circulating concentrations reflect entry into the circulation, distribution, and clearance. None of these measurements, considered in isolation, directly quantifies skeletal muscle IL-15 secretion.

Discordance between these outcomes is consistent with the multilevel regulation of IL-15 biogenesis. Increased *IL15* mRNA abundance need not produce a proportional increase in tissue protein content, and increased tissue protein content does not establish enhanced secretion, because IL-15 may be retained intracellularly or presented in a membrane-associated form. Differences in the timing of transcription, protein accumulation, and release further complicate comparisons. Consequently, an early increase in circulating IL-15 followed by a later rise in muscle *IL15* mRNA abundance does not, by itself, demonstrate that the circulating response originated from de novo synthesis in skeletal muscle.

Circulating IL-15 should not be assumed to originate exclusively from skeletal muscle. Immune and other non-muscle cells may contribute to the circulating pool, while changes in distribution, clearance, and plasma volume may influence measured concentrations. Their contributions to individual exercise protocols remain to be established. An exercise-associated increase alone therefore does not identify cytokine origin, quantify skeletal muscle secretion, or demonstrate an endocrine skeletal muscle–kidney signaling axis. Establishing such an axis requires source-specific evidence, characterization of circulating molecular forms, and demonstration of a receptor-dependent response in renal target cells. Demonstrating renoprotection additionally requires a measurable beneficial renal outcome.

### 3.3. Systemic Versus Intrarenal IL-15 Signaling

Serum or plasma IL-15 concentrations alone do not identify its tissue of origin, distinguish among its molecular forms, or demonstrate biological activity in renal target cells. Exercise-associated extracellular vesicles (EVs) represent another potential route of interorgan communication [141,142]. However, the studies cited here do not establish EV-mediated delivery of biologically active IL-15 from skeletal muscle to the kidney. Testing this mechanism requires establishing the cellular origin of the vesicles, distinguishing EV-associated IL-15 from co-isolated soluble IL-15 or IL-15-containing protein complexes, and demonstrating an IL-15-dependent response in recipient cells [143].

The presence of IL-15 in the circulation does not necessarily imply access to renal target cells. Glomerular filtration into the urinary space should not be equated with delivery to all renal cell populations; access to receptor-bearing cell surfaces and functional receptor engagement must be demonstrated separately. Within the kidney, TECs produce IL-15 and express components of its receptor system, supporting local signaling mechanisms associated with cell survival and preservation of the epithelial phenotype in experimental models [6,38]. Podocytes also respond to IL-15. Separate studies have implicated STAT5 in the maintenance of podocyte autophagic flux and protection against injury and have shown that IL-15 promotes OPA1 expression, mitochondrial fusion, and mitochondrial respiration under diabetic conditions [7,22]. Responses to exogenous IL-15, however, do not by themselves establish endogenous IL-15 production by podocytes or an autocrine signaling mechanism.

Together, these findings support local intrarenal IL-15 signaling and renal responsiveness to IL-15. Separate evidence implicates IL-15 in muscle–kidney communication in experimental DKD. However, these observations do not establish an exercise-induced endocrine pathway. Establishing such a pathway requires source-specific tracing, characterization of circulating IL-15 forms, demonstration of delivery to renal target cells and receptor-dependent signaling, and tissue-specific perturbation of IL-15 production or signaling. Because CD122 is shared by IL-2 and IL-15, and γc participates in several cytokine receptor complexes, activation or disruption of these components alone does not establish IL-15 specificity. IL-15-specific interventions must therefore be combined with source-specific approaches to distinguish the effects of muscle-derived IL-15 from local intrarenal signaling and indirect systemic responses.

## 4. IL-15 Signaling Pathways and Functional Effects in the Kidney

### 4.1. IL-15 Signaling and Cytoprotective Effects in Renal Tubular Epithelial Cells

In renal tissue, IL-15 may contribute to the autocrine and paracrine regulation of tubular epithelial functions. PTECs produce IL-15, express components of its receptor system, and respond to IL-15 stimulation [6]. The tubular epithelium can therefore serve as both a source and a target of IL-15. However, evidence of receptor expression and signaling responses should be distinguished from functional evidence of cytoprotection, particularly when extrapolating experimental findings to the human kidney in vivo.

IL-15 signaling in the tubular epithelium. Primary human PTECs have been shown to express IL-15Rα, CD122, and γc [6]. IL-15Rα supports high-affinity cytokine binding and presentation, whereas CD122 and γc form the canonical signaling module. Depending on receptor composition, cytokine concentration, exposure duration, and mode of presentation, IL-15 can activate JAK/STAT and MAPK/ERK pathways, with additional PI3K/Akt-associated responses also reported in selected experimental systems [8,38,144]. These pathways constitute distinct but potentially interacting signaling branches rather than a single linear cascade.

In primary human PTECs, recombinant human IL-15 induced ERK1/2 and IκBα phosphorylation at 10 and 40 min after stimulation [6]. These findings are consistent with early MAPK/ERK activation and engagement of NF-κB-associated signaling. However, IκBα phosphorylation alone does not establish NF-κB-dependent transcriptional activity. IκBα degradation and p65/RelA nuclear translocation would provide complementary evidence of pathway activation, whereas confirmation of NF-κB-dependent transcription would require additional functional readouts [145].

IL-15 also induced STAT5 phosphorylation in normal PTECs but not in the renal carcinoma cells examined, in which γc and JAK3 proteins were not detected [5]. These findings indicate that receptor composition and cellular phenotype can influence IL-15 responsiveness. Importantly, γc blockade and pharmacological inhibition of JAK3 or STAT5 counteracted IL-15-mediated preservation of E-cadherin in PTECs, supporting a role for this signaling axis in maintaining the epithelial phenotype.

IL-15-responsive signaling has also been investigated during the differentiation of renal cancer stem cells. However, findings from these transformed cells should be considered separately from those obtained in normal tubular epithelium and cannot, on their own, establish physiological signaling mechanisms in the kidney.

Cytoprotective effects. Functional evidence for IL-15-mediated tubular protection derives primarily from cell-culture studies and experimental animal models. Interpretation of these studies should distinguish direct effects on epithelial survival from changes in inflammation or overall renal injury. In experimental nephritis, global IL-15 deficiency increased tubular apoptosis, inflammatory cell accumulation, and renal injury [20]. Complementary experiments in primary murine TECs demonstrated increased susceptibility to apoptosis in IL-15-deficient cells and following blockade of endogenous IL-15, whereas recombinant IL-15 reduced apoptosis in IL-15-deficient cells [20]. These findings support an endogenous survival function for IL-15 in the systems examined. Nevertheless, global IL-15 deletion does not isolate the contribution of tubular epithelial cell-derived IL-15 to whole-kidney outcomes [20].

In a model of cisplatin-induced AKI, intrarenal IL-15 levels decreased 24 h after cisplatin administration. Although this observation does not establish whether the decrease contributes to injury or results from it, the same study provided functional evidence of protection: recombinant IL-15 reduced cisplatin-induced apoptosis in cultured renal epithelial cells, whereas IL-15Rα-deficient renal cells exhibited greater susceptibility to cisplatin-induced apoptosis [144]. Together, these studies indicate that IL-15 can support tubular epithelial survival in defined experimental settings. Whether endogenous or therapeutically administered IL-15 provides comparable protection in the human kidney in vivo remains unresolved.

In primary human proximal tubular epithelial cells, IL-15 preserved E-cadherin expression under culture conditions promoting epithelial dedifferentiation [6]. This finding supports preservation of the epithelial phenotype but does not, by itself, establish reduced tissue fibrosis.

Additional evidence comes from the unilateral ureteral obstruction (UUO) model. Treatment with IL-15, either alone or complexed with IL-15Rα-Fc, reduced MCP-1 expression and collagen and fibronectin accumulation. Macrophage infiltration was significantly reduced by the IL-15/IL-15Rα-Fc complex, whereas IL-15 alone showed a nonsignificant reduction. Neither treatment significantly altered markers of myofibroblast accumulation [21]. Complementary experiments in primary renal myofibroblast cultures demonstrated suppression of TGF-β1-induced collagen and fibronectin production [21]. These results support direct effects on matrix-producing cells alongside modulation of the inflammatory environment, although the relative contributions of these mechanisms to the whole-kidney response remain incompletely defined. Thus, the experimental antifibrotic effects of IL-15 may involve preservation of the epithelial phenotype, reduced extracellular matrix production, and altered inflammatory responses, with outcomes depending on the model, responding cell population, cytokine formulation, and exposure conditions.

Interaction with immune cells. IL-15 regulates the survival, proliferation, and activation of NK cells and CD8^+^ T lymphocytes. Its renal effects may therefore involve immune responses in addition to direct actions on epithelial cells. In human TECs, IFN-γ stimulation and CD40 ligation by CD40L (CD154) can increase *IL15* transcript abundance and IL-15 protein production, with additive effects when both signals are present [146]. Activated T lymphocytes may provide these signals through IFN-γ secretion and CD154-mediated cell contact. The resulting increase in IL-15 production, however, does not establish whether the net tissue response is protective or inflammatory.

In co-cultures of primary human TECs and peripheral blood mononuclear cells (PBMCs), IL-15 blockade inhibited PBMC proliferation, supporting a functional role for IL-15 in epithelial-immune communication [106]. This finding demonstrates an IL-15-dependent proliferative response under the specified culture conditions rather than direct cytoprotection of renal parenchymal cells.

Soluble IL-15 and trans-presentation. Soluble IL-15, soluble IL-15/IL-15Rα complexes, and membrane-associated IL-15 may differ in their contributions to renal responses. During trans-presentation, IL-15 bound to IL-15Rα on a presenting cell engages CD122/γc on an adjacent responding cell. This mechanism is particularly important for NK-cell and CD8^+^ T-cell homeostasis and activation.

Expression of receptor components and responsiveness to exogenous IL-15 do not establish which mode of presentation predominates in the renal tubulointerstitium. Likewise, endogenous IL-15 production does not, by itself, demonstrate the release of biologically active soluble cytokine. Distinguishing these modes of action requires characterization of membrane-associated IL-15, soluble IL-15, and soluble IL-15/IL-15Rα complexes, together with functional assays. Their relative contributions to epithelial survival and epithelial-immune interactions remain incompletely defined.

### 4.2. Podocytes: IL-15-Responsive Signaling and Protection Under Cellular Stress

Endogenous IL-15 production by podocytes is less well established than that by TECs, which has been more extensively characterized. Detection of IL-15 in glomeruli should therefore be distinguished from evidence of podocyte-specific synthesis. Shinozaki et al. (2002) detected IL-15 protein in the tubular and glomerular compartments of normal mouse kidneys and reported reduced expression during nephrotoxic serum nephritis [20]. These observations do not establish which glomerular cell populations synthesize IL-15.

Although NF-κB activation could provide a mechanistic link between inflammatory stimuli and *IL15* transcription, the studies discussed here do not establish direct NF-κB-dependent regulation of *IL15* in podocytes. Evidence of NF-κB pathway activation should therefore not be interpreted as evidence of IL-15 induction or IL-15-dependent cytoprotection.

Podocytes express components of the IL-15 receptor system and respond to exogenous IL-15. Luque et al. (2017) demonstrated IL-15-induced activation of JAK1, JAK3, and spleen tyrosine kinase (SYK) in primary cultured podocytes, with impaired responses in the absence of IL-2/IL-15Rβ (CD122) or the common γ-chain (γc, CD132) [105]. These findings support a functional role for the CD122/γc receptor components in IL-15-induced signaling but do not establish that IL-15Rα is dispensable for all podocyte responses or modes of cytokine presentation.

In the same study, anti-glomerular basement membrane (anti-GBM) glomerulonephritis was induced in *Rag2*−/−, *Rag2*−/−*Il2rg*−/−, and *Rag2*−/−*Il2rb*−/− mice. Bone marrow replenishment experiments supported a protective contribution of glomerular γc during disease [105]. However, these models do not isolate podocyte-specific receptor function. Moreover, because CD122 and γc participate in other cytokine receptor systems, their deficiency alone does not establish IL-15-specific mediation.

The JAK/SYK responses described by Luque et al. [105] and the STAT5 responses examined in a subsequent study were investigated separately and do not establish a single linear signaling cascade responsible for the observed protective effects [7]. In cultured human podocytes, exogenous IL-15 increased STAT5B phosphorylation after 30 min of stimulation [7]. Complementary genetic experiments demonstrated a functional contribution of STAT5 to podocyte stress resistance. Mice with *Nphs2*-Cre-mediated podocyte-specific deletion of *Stat5a* and *Stat5b* exhibited more severe proteinuria and glomerular abnormalities in experimental adriamycin nephropathy and anti-GBM glomerulonephritis. Separately, deletion of *STAT5B* in cultured human podocytes reduced autophagic flux. These findings support a protective role for podocyte STAT5 and its involvement in autophagic regulation, without establishing that these functions depend exclusively on IL-15.

In a mouse model of adriamycin-induced focal segmental glomerulosclerosis (FSGS), administration of an IL-15/IL-15Rα complex 24 h before and on days 1, 3, and 5 after adriamycin injection reduced albuminuria and podocyte ultrastructural abnormalities [7]. Treatment also reduced glomerular p62/SQSTM1 accumulation, a finding compatible with improved autophagic clearance but insufficient, on its own, to demonstrate increased autophagic flux. The number of renal T cells also increased, leaving the relative contributions of direct effects on renal parenchymal cells and immune-mediated effects incompletely resolved.

Together with the podocyte-specific Stat5 deletion experiments, these findings support a protective role for podocyte STAT5 and beneficial effects of IL-15/IL-15Rα treatment in the studied model. However, they do not establish that the treatment effects are mediated exclusively through podocyte STAT5. STAT5B-deficient cultured human podocytes also showed a trend toward reduced TOM20 abundance, which was used as a proxy for mitochondrial mass. This observation does not, by itself, establish mitochondrial depletion or dysfunction [7].

The evidence should therefore be interpreted within the context of each experimental system. Human biopsy findings demonstrate nuclear accumulation of phosphorylated STAT5B in podocytes in FSGS. Studies in cultured human podocytes demonstrate IL-15 responsiveness and functional consequences of STAT5B deletion, whereas animal studies demonstrate protection under defined injury conditions. These findings do not establish that endogenous or therapeutically administered IL-15 prevents glomerular injury in humans in vivo.

Metabolic stress may also regulate the podocyte IL-15 receptor system. Li et al. (2026) reported increased IL-15Rα expression in WT1-positive podocytes from diabetic mice, increased *Il15ra* transcript levels in cultured mouse podocytes exposed to high glucose, and a concordant increase in *Il15ra* expression in a single-cell dataset from diabetic mouse kidneys [22]. These observations support context-dependent receptor regulation but do not, by themselves, establish enhanced IL-15 signaling or protection in human DKD.

In the same study, IL-15-dependent mitochondrial regulation was investigated in a model of muscle-kidney communication [22]. Skeletal muscle-specific PGC-1α overexpression was used to generate diabetic mPGC-1α:db/db mice, and recombinant murine IL-15 was separately administered to db/db mice. Increased skeletal muscle IL-15 mRNA and protein expression and elevated plasma IL-15 concentrations accompanied improved renal outcomes in the muscle-specific transgenic model. In cultured mouse podocytes, IL-15 increased OPA1 expression, preserved a more filamentous mitochondrial network under high-glucose conditions, and improved basal, ATP-coupled, and maximal respiration. OPA1 knockdown abolished the protective effect of IL-15 on mitochondrial morphology and reduced nephrin and COX IV abundance despite IL-15 supplementation, supporting a functional requirement for OPA1 in these responses.

These results provide evidence for a potential muscle-kidney mechanism in experimental DKD but do not establish physiological exercise-associated delivery of muscle-derived IL-15 to podocytes. Genetic enhancement of muscle PGC-1α and administration of recombinant IL-15 are distinct interventions, and other circulating factors may contribute to the renal effects of the muscle-specific transgenic model.

IL-15 effects on glomerular injury are not limited to direct actions on podocytes. In a separate study, anti-IL-15 or anti-CD122 treatment attenuated albuminuria, podocyte injury, and glomerular lesions in adriamycin-treated nude mice following adoptive transfer of CD8^+^CD69^−^CD44^+^ memory T cells, while reducing renal CD8^+^ tissue-resident memory T (TRM)-cell formation and activation. Similar protective effects of IL-15 signaling blockade were observed in db/db mice. Separate NK-cell depletion experiments did not alleviate podocyte or glomerular injury in adriamycin-treated or db/db mice, supporting the contribution of CD8^+^ T-cell rather than NK-cell responses in these experimental settings. These immune-mediated mechanisms should be distinguished from direct parenchymal responses and should not be generalized across all kidney diseases [147].

Thus, IL-15-responsive mechanisms may have opposing consequences across experimental settings, including different studies of diabetic kidney injury. Receptor-associated kinase responses and STAT5-associated protection in podocytes coexist with IL-15-driven formation and activation of pathogenic renal CD8^+^ TRM cells. The available evidence does not establish a universal “low-dose protective/high-dose harmful” relationship. The balance between protective and injurious effects may depend on cytokine source and availability, exposure duration, presentation mode, receptor composition, and inflammatory context.

### 4.3. Regulation of IL-15R Subunit Expression in Renal Cells Under Pathological Conditions

Experimental studies indicate that expression of IL-15 receptor components can vary with cell type and pathological conditions [6,8,105]. These changes should be distinguished from alterations in cytokine availability and from evidence of functional receptor-dependent responses.

In TECs, IL-15 has been shown to support cell survival in experimental systems [20]. In primary human TECs, IFN-γ increased IL-15Rα expression, whereas IL-2 and IL-15 did not produce this effect [8]. This finding demonstrates inflammatory regulation of IL-15Rα expression but does not establish that IFN-γ-induced upregulation enhances IL-15 signaling or cytoprotection.

Changes in cytokine availability provide a related but distinct observation. In experimental sepsis-associated renal injury, ischemia–reperfusion injury, and cisplatin nephrotoxicity, intrarenal IL-15 levels decreased. Functional experiments in the same study showed that recombinant IL-15 reduced cisplatin-induced apoptosis in cultured renal epithelial cells, whereas IL-15Rα-deficient renal epithelial cells exhibited greater cisplatin-induced apoptosis [144]. These findings support a contribution of IL-15Rα to epithelial survival under the conditions studied, but reduced tissue IL-15 levels alone do not establish whether cytokine loss is a cause or consequence of renal injury.

Whole-kidney *Il15* and *Il15ra* transcript levels were lower in obstructed than in contralateral kidneys on day 8 after unilateral ureteral obstruction (UUO) [21]. These tissue-level measurements do not identify the cell populations responsible for the changes.

The glomerular compartment provides additional evidence of receptor regulation. Increased γc expression was detected on podocytes in human glomerulonephritis, and experimental podocyte responses to IL-15 involved CD122 and γc [105]. However, increased receptor expression alone does not establish greater functional responsiveness, and the shared use of these subunits by other cytokines limits conclusions about IL-15 specificity. A subsequent study demonstrated IL-15-induced STAT5B phosphorylation and, in complementary genetic experiments, a functional contribution of STAT5 to podocyte autophagic flux and stress resistance [7]. These downstream findings do not establish how pathological changes in receptor subunit expression affect IL-15 responsiveness.

Metabolic stress may also regulate receptor expression. Li et al. (2026) reported increased IL-15Rα protein expression in WT1-positive podocytes from diabetic mice, supported by immunoblotting of isolated podocytes [22]. High-glucose exposure increased *Il15ra* transcript levels in cultured mouse podocytes, and increased podocyte *Il15ra* expression was also identified in a mouse diabetic-kidney single-cell dataset [22]. These observations support regulation of IL-15Rα under diabetic conditions but do not, by themselves, establish enhanced signaling or protection in human diabetic kidney disease.

Overall, these studies demonstrate context-dependent regulation of individual IL-15 receptor components rather than a uniform pattern of receptor regulation across renal cell types and disease models. In the canonical IL-15 receptor system, IL-15Rα contributes to high-affinity cytokine binding, stabilization, and presentation, whereas CD122 and γc form the principal signaling module. Changes in receptor abundance may therefore affect cytokine handling and cellular responsiveness in different ways, depending on receptor composition and the mode of IL-15 presentation. Their functional consequences require direct assessment.

The tubular epithelium is both a source of IL-15 and a cytokine-responsive compartment. In podocytes, responsiveness to IL-15 is better established than endogenous cytokine production. Direct parenchymal responses should also be distinguished from immune-cell effects on lymphocyte survival, proliferation, and activation. Receptor expression alone cannot determine the net balance between these responses.

Whether comparable changes in receptor expression or IL-15 presentation occur in response to physical exercise remains unresolved. Establishing their relevance to exercise-associated renal stress will require cell-specific measurements of IL-15 availability, receptor expression, and functional signaling, together with experiments distinguishing locally produced renal IL-15 from muscle-derived IL-15 that may be delivered to the kidney.

## 5. Established Exercise-Induced Renal Stress Versus Hypothetical IL-15-Mediated Mechanisms

### 5.1. Clinical Interpretation of Exercise-Associated Renal Stress and the Nonspecific Nature of NF-κB Activation

During intense physical exercise, redistribution of blood flow toward active skeletal muscles can reduce renal perfusion and impose hemodynamic stress on the kidney. The magnitude of this reduction varies with exercise intensity and duration, hydration status, and environmental conditions [148,149]. Reduced renal perfusion may increase susceptibility to regional hypoxia, particularly in the renal medulla, although a reduction in renal blood flow alone does not establish tissue ischemia or structural injury. Exercise-associated renal responses should therefore be evaluated using complementary functional measurements and biomarkers of cellular stress or injury.

Increases in urinary neutrophil gelatinase-associated lipocalin (NGAL), kidney injury molecule-1 (KIM-1), liver-type fatty acid-binding protein (L-FABP), and IL-18 have been reported after strenuous exercise [150,151]. Cystatin C should be interpreted according to the compartment measured: serum cystatin C is primarily used as a filtration marker, whereas increased urinary cystatin C may indicate impaired proximal tubular reabsorption and catabolism [152,153,154]. These measurements are not interchangeable indicators of tubular injury. Post-exercise biomarker elevations are not necessarily accompanied by sustained impairment of kidney function [151,155]. In several studies, urinary biomarker concentrations increased shortly after exercise and declined during recovery, with selected markers returning to baseline within 24–48 h. However, the magnitude and duration of these changes varied across markers, protocols, and study populations [25,151,156]. Neither an isolated biomarker increase nor its rapid resolution is sufficient to distinguish transient renal injury from a physiological response without demonstrable injury.

Post-exercise biomarker changes should be interpreted alongside serial serum creatinine measurements, urine output, where data are available, hydration status, exercise duration and intensity, and relevant clinical risk factors [150]. Conventional creatinine-based estimated glomerular filtration rate (eGFR) should be interpreted cautiously when serum creatinine is changing rapidly and steady-state assumptions are not satisfied [157]. Biomarker changes, evidence of cellular stress or injury, fulfillment of conventional acute kidney injury (AKI) diagnostic criteria, and clinical consequences represent distinct but potentially overlapping levels of assessment.

Under the KDIGO 2012 criteria, AKI is defined by any of the following: an increase in serum creatinine of ≥0.3 mg/dL (≥26.5 μmol/L) within 48 h; an increase in serum creatinine to ≥1.5 times baseline, known or presumed to have occurred within the preceding 7 days; or urine output <0.5 mL/kg/h for at least 6 h. Exercise studies should specify which criterion was met and the timing of baseline and post-exercise assessments. Rapid recovery does not, by itself, exclude an episode of AKI. However, a post-exercise creatinine increase meeting the numerical threshold requires contextual interpretation because changes in creatinine generation and distribution may contribute alongside reduced filtration [158]. The duration of dysfunction, subsequent recovery, and associated complications remain important for clinical interpretation.

Biomarker specificity introduces additional uncertainty. NGAL is produced by renal epithelial cells and extra-renal sources, including activated neutrophils; systemic inflammation may therefore influence circulating concentrations independently of direct renal parenchymal injury [159,160]. Urinary measurements require separate consideration of renal production, tubular handling, and urine concentration. Similarly, post-exercise serum creatinine may be influenced by creatinine generation, muscle injury, and changes in plasma volume, in addition to reduced filtration [23,149]. Dehydration may also contribute to genuine renal hypoperfusion and should not be regarded solely as a confounder. These limitations warrant contextual interpretation of KDIGO criteria rather than an assumption that exercise-associated increases necessarily represent false-positive AKI. Serial measurements and the clinical course are needed both to identify episodes meeting diagnostic criteria and to assess their consequences.

Exertional rhabdomyolysis represents a distinct pathological context in which myoglobin released from damaged skeletal muscle may contribute to kidney injury [161,162]. Filtered myoglobin can be taken up by PTECs, and its heme-associated toxicity may promote oxidative stress, mitochondrial dysfunction, and regulated cell-death pathways, including ferroptosis [163,164,165,166]. In experimental crush-syndrome AKI, RIG-I interacted with myoglobin and contributed to NF-κB/caspase-3-associated apoptotic signaling; RIG-I depletion attenuated myoglobin-induced apoptosis-associated responses [167]. These findings should not be extrapolated directly to exercise without overt rhabdomyolysis.

TECs are important targets of ischemic, hypoxic, toxic, and oxidative injury during AKI [168]. Experimental rhabdomyolysis-associated injury has been accompanied by ATP depletion, increased reactive oxygen species (ROS) production, cytochrome *c* release, and apoptosis [169,170,171]. These findings identify mechanisms observed in experimental rhabdomyolysis-associated injury but do not establish their occurrence or sequence during strenuous exercise without rhabdomyolysis.

NF-κB functions as a broad signaling hub that integrates oxidative, inflammatory, and tissue-injury signals. Its activation is not specific to IL-15 and may occur in response to myoglobin-associated signaling [167], ROS-associated stress [172,173], pro-inflammatory cytokines, and DAMPs [174]. Depending on the experimental system, NF-κB-associated responses in TECs include induction of pro-inflammatory cytokines and chemokines. TWEAK/Fn14 stimulation can also engage noncanonical NF-κB activation involving RelB/p52 and induction of CCL19 and CCL21 in experimental tubular-cell systems [175]. The activated subunits, target genes, and response kinetics depend on the stimulus and cell type; these findings do not establish the same program during exercise.

As discussed in Section 4.1, experimental studies have linked IL-15 to preservation of epithelial features and modulation of extracellular matrix production under defined conditions [6,38]. These findings do not establish NF-κB-dependent IL-15 induction or IL-15-mediated renal protection during exercise.

NF-κB-associated stress responses and IL-15-responsive cellular effects should therefore be treated as distinct evidence streams. NF-κB activation does not, by itself, distinguish adaptation from injury, and NF-κB and IL-15 should not be assigned exclusively injurious and protective roles, respectively. Their consequences depend on the responding cell population, stimulus, signal duration, and inflammatory environment. For IL-15, cytokine availability, presentation mode, receptor composition, exposure duration, and immune-cell involvement may further influence outcomes. The available evidence does not establish a universal low-dose protective/high-dose harmful relationship or a specific NF-κB-dependent threshold separating adaptation from injury during physical exercise.

Accordingly, the sequence “exercise-associated stress → NF-κB activation → intrarenal IL-15 induction → parenchymal protection” remains a conceptual hypothesis rather than an established pathway. The studies discussed here do not demonstrate that exercise-associated renal NF-κB activation increases local IL-15 production or that such production mediates an autocrine or paracrine protective response. Reported increases in circulating IL-15 after selected exercise protocols also do not establish its skeletal muscle origin, renal delivery, or direct action on renal parenchymal cells. Proposed delivery of biologically active IL-15 through muscle-derived extracellular vesicles requires separate validation, as discussed in Section 3.3.

**Figure 2:**
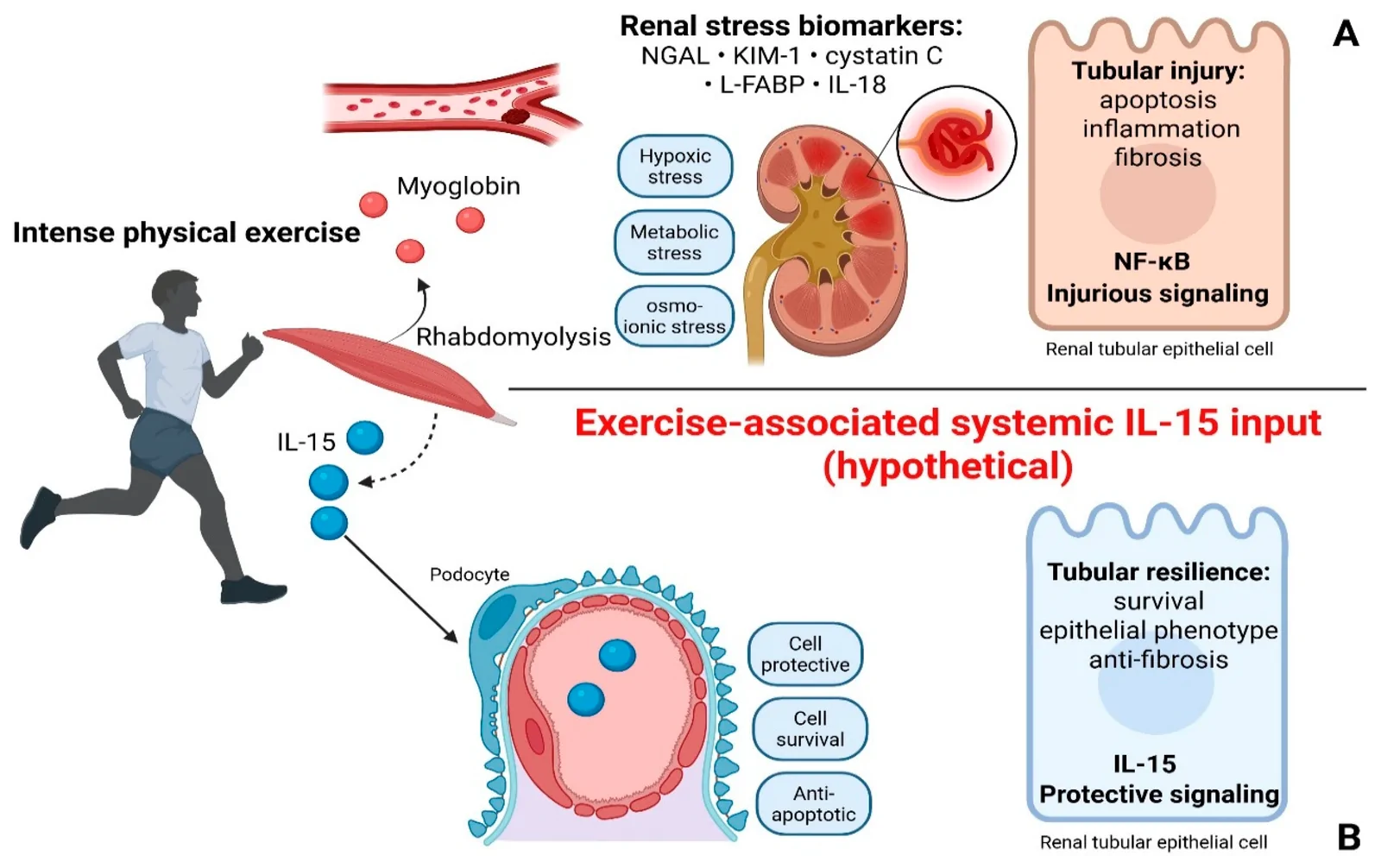
Proposed IL-15-dependent signaling pathways in renal cells during intense physical exercise ((**A**)—renal stress, (**B**)—cytoprotective pathways). (**A**). Intense physical exercise may impose hemodynamic, hypoxic, metabolic, and osmotic or ionic stress on the kidney, potentially resulting in a transient increase in renal stress biomarkers, including NGAL, KIM-1, cystatin C, L-FABP, and IL-18. When exercise-associated rhabdomyolysis occurs, myoglobin released from injured skeletal muscle enters the circulation and may contribute to tubular injury. Myoglobin-associated oxidative stress, mitochondrial dysfunction, and inflammatory signaling may promote NF-κB-associated responses, apoptosis, inflammation, and profibrotic remodeling in renal tubular epithelial cells (TECs). (**B**). Conversely, a transient systemic IL-15 signal associated with exercise may activate cytoprotective pathways in renal cells, supporting cell survival, preservation of the epithelial phenotype, and antifibrotic responses. The origin, renal bioavailability, molecular form, and direct effects of circulating IL-15 during exercise remain hypothetical and require experimental validation. Abbreviations: IL-15, interleukin-15; IL-18, interleukin-18; NGAL, neutrophil gelatinase-associated lipocalin; KIM-1, kidney injury molecule-1; L-FABP, liver-type fatty acid-binding protein; NF-κB, nuclear factor kappa B.

Figure 2 should therefore be interpreted as a conceptual synthesis of exercise-associated renal stress and potentially relevant IL-15-responsive mechanisms identified in other experimental settings, rather than an experimentally established causal pathway. Establishing a connection will require studies that distinguish locally produced renal IL-15 from potential endocrine delivery, measure cell-specific signaling, and combine IL-15-specific genetic or pharmacological interventions with functional renal outcomes.

### 5.2. Evidence Boundaries for the Skeletal Muscle–IL-15–Kidney Axis

The proposed exercise-associated skeletal muscle–IL-15–kidney axis draws on several independently established lines of evidence. Skeletal muscle expresses *IL15*, and selected exercise protocols alter muscle transcript abundance or circulating IL-15 concentrations, although changes in muscle transcript abundance, tissue protein levels, and circulating concentrations do not necessarily coincide. Renal TECs produce IL-15 and exhibit functional responses to IL-15 stimulation [8,20]. Separate podocyte studies have identified IL-15-responsive JAK1/JAK3 and SYK signaling, IL-15-induced STAT5B phosphorylation, a functional contribution of STAT5 to autophagic regulation, and OPA1-associated mitochondrial responses under high-glucose conditions [7,22,95,105]. These findings do not establish a single causal pathway linking exercise-induced muscle IL-15 production to renal protection.

A potential connection between IL-15 signaling and podocyte survival or metabolic regulation warrants further investigation. Nephrin and CD2AP interact with the p85 regulatory subunit of PI3K and, together with podocin, promote PI3K-dependent Akt signaling associated with podocyte survival. However, the studies reviewed here do not establish that IL-15 activates this signaling module in podocytes [176].

IL-15 overexpression has been associated with increased skeletal muscle PGC-1α protein expression and an oxidative metabolic phenotype [177]. However, the studies reviewed here do not establish an equivalent IL-15-responsive program in podocytes. In cultured mouse podocytes exposed to high glucose, IL-15 improved mitochondrial respiration, increased OPA1 expression, and preserved a more filamentous mitochondrial network [22]. Separately, IL-15-induced STAT5B phosphorylation and a functional contribution of STAT5B to autophagic flux were demonstrated in cultured human podocytes [7]. These findings identify potentially relevant mechanisms of podocyte stress resistance, but they do not establish exercise-induced activation of these mechanisms or a direct effect of IL-15 on podocyte mitochondrial biogenesis.

The studies reviewed here do not establish whether exercise generates a circulating pool of biologically active, muscle-derived IL-15 that reaches renal target cells and activates IL-15 receptor pathways in TECs or podocytes. Changes in circulating concentrations alone cannot establish the cytokine source, molecular form, renal exposure, or functional receptor engagement.

The balance between direct parenchymal and immune-mediated IL-15 effects also requires exercise-specific investigation. Cytokine availability, exposure duration, presentation mode, receptor composition, and inflammatory context may influence the responding cell populations and functional outcomes. The available evidence does not establish that short-term local signaling is necessarily protective or that prolonged systemic exposure is necessarily injurious.

Accordingly, the proposed exercise-associated skeletal muscle–IL-15–kidney axis remains a biologically plausible but unproven interorgan model. Table 3 summarizes the evidence supporting individual proposed steps and the principal limitations of extrapolating these findings to physical exercise.

### 5.3. Context-Dependent Renal IL-15 Signaling: Parenchymal Protection and Immune-Mediated Injury

The functional consequences of IL-15 signaling depend on cytokine availability, exposure duration, mode of presentation, receptor composition, responding cell populations, and the inflammatory state of the tissue microenvironment. These factors provide a framework for interpreting different experimental outcomes but do not establish a universal dose- or duration-dependent switch between protection and injury.

In renal epithelial systems, IL-15 can support cell survival and preservation of the epithelial phenotype under defined experimental conditions [6]. IL-15-responsive pathways include JAK/STAT, MAPK/ERK, and PI3K/Akt signaling, although their relative involvement and functional significance depend on the cell type and stimulation conditions [178]. These findings support potential protective effects on renal parenchymal cells but do not demonstrate that physiological or transient IL-15 exposure is invariably beneficial.

Podocyte experiments have demonstrated a functional contribution of STAT5 to autophagic flux and stress resistance. Separately, treatment with an IL-15/IL-15Rα complex produced beneficial effects in experimental FSGS [7]. Other work linked IL-15 to OPA1-associated mitochondrial fusion and respiratory function in experimental DKD [22]. These mechanisms were investigated in different settings and should not be combined into a single established protective pathway. Whether comparable responses occur during physical exercise remains unresolved.

IL-15 also supports the survival, expansion, and activation of NK cells and memory CD8^+^ T-cell populations through presentation-dependent mechanisms [178]. Such responses may contribute to normal immune homeostasis or, in an inflammatory setting, sustain injurious cytotoxic activity. Relevant outputs include the production of inflammatory cytokines such as IFN-γ and cytotoxic effector molecules such as granzyme B and perforin. However, their induction alone does not establish renal tissue injury.

Evidence for an IL-15-dependent, injurious renal immune response comes from experiments in adriamycin nephropathy, in which blockade of IL-15 signaling attenuated podocyte injury and glomerulosclerosis by inhibiting renal CD8^+^ TRM-cell responses rather than suppressing NK cells. This mechanism should be distinguished from direct IL-15-responsive signaling in renal parenchymal cells and should not be generalized to all kidney diseases.

Oxidative stress, damage-associated molecular patterns, and NF-κB-associated inflammatory responses may influence the environment in which IL-15 acts. However, these conditions do not demonstrate that IL-15 necessarily drives epithelial apoptosis or fibrosis. Indeed, treatment with either IL-15 alone or an IL-15/IL-15Rα complex reduced extracellular-matrix accumulation in experimental unilateral ureteral obstruction, illustrating that inflammatory or fibrotic injury does not predict an exclusively harmful IL-15 response [21].

**Figure 3:**
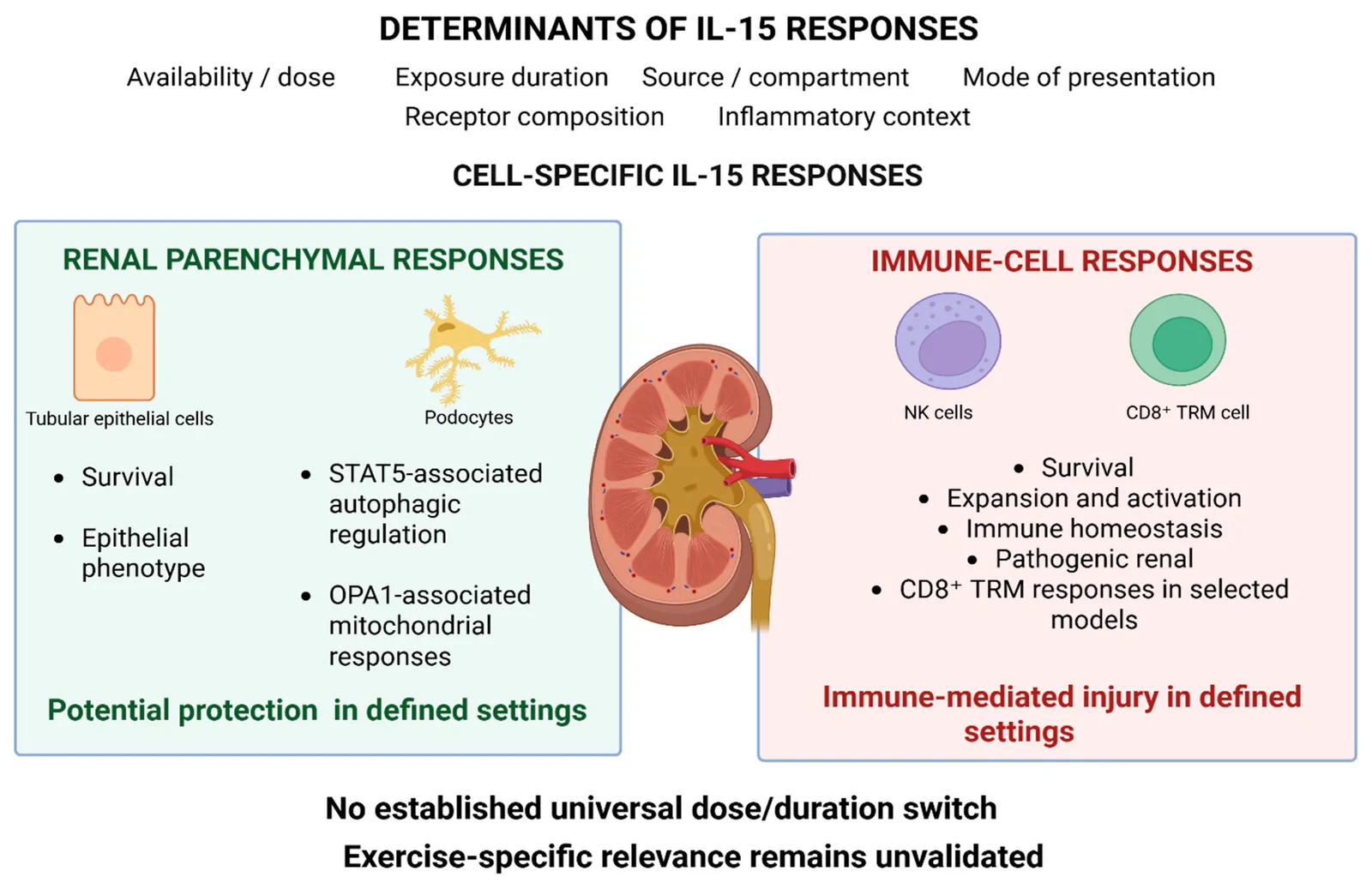
Context-dependent renal effects of IL-15: parenchymal protection and immune-mediated injury.

Thus, the net renal outcome cannot be inferred from cytokine concentration alone or described by a universal low-dose protective/high-dose harmful model. Experimental studies identify beneficial parenchymal responses and IL-15-driven formation and activation of pathogenic renal CD8^+^ TRM cells in distinct settings. The relative contributions of these effects depend on the experimental context and require direct assessment; their temporal dynamics and relevance to exercise-associated renal stress remain unresolved. These context-dependent responses are summarized in Figure 3.

The consequences of IL-15 signaling depend on cytokine availability and dose, exposure duration, cellular source and compartment, mode of presentation, receptor composition, and the inflammatory context. Experimental studies in renal parenchymal cells support effects on tubular epithelial survival and phenotype preservation, STAT5-associated autophagic regulation and stress resistance in podocytes, and OPA1-associated mitochondrial responses under diabetic conditions. These findings derive from distinct experimental systems and do not establish a single integrated protective pathway. In the immune compartment, IL-15 supports NK-cell and memory CD8^+^ T-cell survival, expansion, and activation. These responses may contribute to immune homeostasis, whereas pathogenic renal CD8^+^ tissue-resident memory T-cell responses have been implicated in injury in selected disease models. The two branches represent potentially coexisting responses rather than mutually exclusive states. Dose and exposure duration are shown as contextual determinants, not as established thresholds separating protection from injury. A universal low-dose protective/high-dose harmful relationship has not been demonstrated, and the relevance of these mechanisms to exercise-associated renal stress remains to be validated.

### 5.4. Methodological Framework for Validating the Skeletal Muscle–IL-15–Kidney Axis

To advance the proposed skeletal muscle–IL-15–kidney communication pathway beyond biological plausibility and establish it as a physiological mechanism, future investigations should integrate complementary molecular, cellular, and whole-organism approaches. Measurements confined to a single biological compartment or endpoint, such as circulating cytokine concentrations or renal injury markers assessed in isolation, are insufficient to establish causal interorgan signaling. A comprehensive validation strategy should therefore combine evidence of (1) exercise-associated changes in skeletal muscle IL-15 production and release, (2) systemic availability of biologically active muscle-derived IL-15, (3) renal delivery and receptor engagement, and (4) functional consequences for renal stress, injury, or recovery. The following criteria constitute a proposed framework for future validation studies.

Criterion 1: Assessment of skeletal muscle IL-15 production and release during exercise.

Future studies should first establish whether the exercise stimulus alters the skeletal muscle IL-15 system. This requires quantification of *IL15* and *IL15RA* transcripts, together with assessment of the abundance and cellular localization of the corresponding proteins, at appropriately selected time points before, during, and after exercise. Where feasible, cell-resolved analyses should distinguish expression in myofibers from that in resident or infiltrating immune cells and other non-myofiber populations. These measurements characterize the muscle IL-15 system but do not, by themselves, establish cytokine release. Evidence of release requires complementary source-resolved measurements that distinguish changes in tissue abundance from the appearance of IL-15 in the extracellular compartment and its subsequent systemic availability.

These analyses may include reverse transcription quantitative polymerase chain reaction (RT-qPCR) for *IL15* and *IL15RA* transcripts, together with validated methods for detecting IL-15 and IL-15Rα proteins, such as immunoblotting, immunoassays, or targeted proteomic approaches. Species-appropriate gene nomenclature should be used, including *Il15* and *Il15ra* in mouse models. Because IL-15 availability is regulated by intracellular trafficking and receptor-associated presentation, analysis of relevant transcript variants and intracellular localization patterns may provide additional insight into the mechanisms controlling cytokine availability.

Criterion 2: Characterization of circulating IL-15 molecular forms and biological activity.

An exercise-associated increase in circulating IL-15 does not, by itself, establish endocrine communication, because concentration measurements alone do not identify the cytokine source, molecular form, or biological activity. Future studies should distinguish uncomplexed soluble IL-15 from soluble IL-15/IL-15Rα complexes using appropriately validated assays. Sampling time, sample handling, and exercise-associated changes in plasma volume should also be considered.

IL-15Rα-dependent stabilization and presentation should be distinguished from signaling through CD122 and γc in responding cells. Molecular characterization should therefore be complemented by functional assays incorporating controls that establish whether the observed response is IL-15-dependent. EVs represent another potential route of interorgan communication, but the studies discussed here do not establish that muscle-derived EVs transport biologically active IL-15. Where this mechanism is proposed, studies should establish vesicle origin, distinguish EV-associated cytokine from co-isolated soluble proteins, and demonstrate an IL-15-dependent response in recipient cells. Appropriate non-vesicular fractions and EV-depleted samples should also be included as controls to determine whether the observed response depends on EV-associated IL-15 rather than co-isolated soluble cytokines.

Criterion 3: Direct assessment of renal delivery and target-cell accessibility.

A critical unresolved question is whether endogenous IL-15 originating from skeletal muscle reaches renal target cells during exercise. Future studies should use source-specific tracing approaches that distinguish between cytokine production, release, and delivery to renal tissue. Genetically encoded expression reporters may identify producing cells, but they do not, by themselves, track secreted IL-15.

Administration of labeled IL-15 or IL-15/IL-15Rα complexes may provide information about distribution and renal accessibility, but it does not establish physiological muscle-to-kidney transfer. Such experiments should verify that labeling preserves cytokine activity and distinguish tissue-associated signal from residual intravascular material or nonspecific uptake. Localization within renal compartments must also be distinguished from access to receptor-bearing surfaces on specific target cells and from functional receptor engagement.

Criterion 4: Demonstration of renal IL-15 receptor engagement and downstream signaling.

Detection of IL-15 or IL-15/IL-15Rα complexes within renal tissue is insufficient to establish functional signaling. Future studies should characterize the abundance and cellular localization of IL-15 receptor components, including IL-15Rα, CD122, and γc, and examine receptor-associated signaling in the relevant responding cells. Downstream signaling should be evaluated using context-appropriate readouts, such as phosphorylation of JAK1, JAK3, and STAT5, with additional analysis of Akt, ERK1/2, or SYK where justified by the cellular model. These signaling components should not be assumed to constitute a single linear cascade or to contribute equally to each functional outcome. Because activation of these signaling components is not specific to IL-15, attribution of the observed responses to IL-15 requires IL-15-specific perturbation and appropriate controls rather than phosphorylation measurements alone. Cell-specific approaches, including validated cell-isolation methods, spatial identification, or lineage-based analyses of tubular epithelial cells and podocytes, will be needed to distinguish renal parenchymal responses from signals originating in immune cells.

Criterion 5: Linking IL-15 signaling to renal function, injury, and recovery.

Establishing a functional skeletal muscle–IL-15–kidney axis requires evidence that receptor engagement produces measurable biological consequences. Future studies should integrate molecular analyses with serial measurements of renal function and urinary stress- or injury-associated biomarkers, including NGAL, KIM-1, L-FABP, and IL-18. Serum creatinine and urine output should be interpreted alongside hydration status, muscle injury, and the clinical or experimental context. Conventional creatinine-based eGFR should be interpreted cautiously when serum creatinine is changing rapidly and steady-state assumptions are not satisfied. In experimental models, genetic or pharmacological perturbation of IL-15 should be linked to relevant cellular outcomes, such as tubular apoptosis, podocyte architecture, autophagic flux, slit-diaphragm protein integrity, mitochondrial fusion, and mitochondrial respiratory function. Studies should evaluate protective, neutral, and potentially injurious outcomes rather than presume a protective effect. Changes in biomarker levels, AKI defined by established diagnostic criteria, structural injury, and recovery should be evaluated as related but distinct endpoints.

Criterion 6: Testing causality through tissue-specific genetic models.

Because IL-15 is produced by multiple tissues and acts on immune and parenchymal populations, strong causal attribution requires source- and target-cell-resolved approaches. Tissue-specific genetic models provide one strategy for addressing this requirement. Myofiber-specific deletion of *Il15* would test the contribution of myofiber-derived IL-15 to circulating IL-15 levels and renal responses during exercise. Such an intervention would not, by itself, assess IL-15 production by other cells within skeletal muscle tissue. Myofiber-specific perturbation of the gene encoding IL-15Rα would instead allow assessment of the contribution of receptor-associated stabilization and presentation; these interventions should not be treated as equivalent.

Target-cell-specific perturbation of relevant receptor or signaling components would test whether the observed renal outcomes depend on direct signaling in tubular epithelial cells or podocytes. However, disruption of CD122 or γc alone cannot establish IL-15 specificity and should be combined with IL-15-specific interventions.

Studies should verify targeting efficiency and cellular specificity, include appropriate control groups, and assess systemic immune effects as well as changes in exercise capacity or workload. Where feasible, inducible models and rescue experiments may strengthen causal interpretation and help distinguish acute signaling effects from developmental consequences. Rescue experiments should aim to restore exposure within a physiologically relevant range, because rescue with pharmacological cytokine doses would not, by itself, validate the endogenous exercise-associated mechanism.

## 6. Evidence Limitations and Methodological Caveats

Several methodological limitations and evidence gaps constrain validation of an exercise-associated skeletal muscle–IL-15–kidney axis. These limitations concern translation from experimental models to human physiology and extrapolation from kidney disease to acute exercise. In addition, the studies reviewed lack direct, source-specific evidence linking muscle-derived IL-15 to renal responses during exercise.

Translational gaps between experimental models and human physiology. Much of the mechanistic evidence concerning the effects of IL-15 on cell survival, autophagy, and mitochondrial regulation derives from cultured renal cells and experimental animal models [7,22]. These approaches have identified functional responses and signaling mechanisms, but the interventions used—including gene deletion, administration of recombinant cytokine or cytokine-receptor complexes, and genetic enhancement of muscle metabolic programs—differ from endogenous cytokine exposure during human exercise.

Such differences limit physiological extrapolation but do not invalidate the functional evidence obtained in the original experimental setting. For example, podocyte-specific deletion of *Stat5a* and *Stat5b* in mice and *STAT5B* deletion in cultured human podocytes support a contribution of STAT5 to stress resistance and autophagic regulation [7]. Separately, effects of IL-15 on mitochondrial fusion and respiratory function have been demonstrated under diabetic conditions [22]. These results support specific signaling and functional relationships within defined systems, not a single integrated pathway operating during exercise. Observations in human tissues likewise need to be distinguished from evidence of functional protection obtained in cell cultures or animals.

Constraints on extrapolation from kidney disease to acute exercise. The disease context is an additional limitation. Evidence supporting a potential IL-15-mediated muscle-kidney mechanism has been obtained in experimental DKD, where persistent hyperglycemia and associated mitochondrial and inflammatory abnormalities shape the renal response. Li et al. (2026) investigated skeletal muscle-specific PGC-1α overexpression and conducted separate experiments with recombinant IL-15, linking IL-15 to OPA1-associated mitochondrial fusion and improved podocyte respiratory function [22]. These interventions and outcomes should not be equated with the effects of a physiological exercise bout.

Other studies have examined the protective effects of IL-15 in experimental renal injury and fibrogenesis, including nephrotoxic serum nephritis and unilateral ureteral obstruction [21,107]. Although these findings provide a rationale for investigating renal adaptation, the initiating stimuli, duration of the experimental challenge, immune environment, and baseline tissue state differ from those encountered during exercise.

Acute exercise may alter renal perfusion, hydration, redox balance, and inflammatory responses, but the magnitude and time course of these changes depend on the exercise protocol and individual risk factors [24,179,180]. Consequently, disease-model findings alone cannot establish whether IL-15-responsive mechanisms are engaged during exercise or whether their net effects are protective, neutral, or injurious. Transient biomarker changes should also not be assumed to represent harmless adaptation without assessment of renal function and evaluation of the subsequent clinical or experimental course.

Gaps in direct source tracing and in vivo validation in humans. The exercise studies reviewed here do not demonstrate that endogenous IL-15 originating from skeletal muscle reaches defined renal target cells in humans and activates receptor-dependent signaling [35,95]. Increased circulating concentrations do not establish muscle secretion, because immune and other non-muscle cells may contribute to the cytokine pool. Muscle transcript abundance, tissue protein content, and circulating concentrations must therefore be interpreted as distinct measurements.

Changes in plasma volume, cytokine distribution, and clearance may also influence circulating concentrations, although their specific contributions to IL-15 responses require evaluation within individual exercise protocols. Human exercise studies have predominantly measured circulating IL-15 rather than directly traced its tissue source and renal delivery [35]. Furthermore, routine concentration measurements do not necessarily distinguish uncomplexed IL-15 from soluble IL-15/IL-15Rα complexes or establish biological activity in renal cells. EV-mediated transport remains a separate hypothesis requiring verification of vesicle origin, cytokine association, and an IL-15-dependent functional response. Receptor expression, tissue localization, and phosphorylation of shared signaling proteins are likewise individually insufficient to establish an endocrine mechanism.

Consequently, the proposed exercise-associated skeletal muscle–IL-15–kidney axis should currently be regarded as a biologically plausible working hypothesis rather than an experimentally verified physiological mechanism. Validation requires source-resolved in vivo studies demonstrating renal target-cell exposure, IL-15-dependent signaling, and functional renal consequences. EV tracking is required only where vesicular transport is proposed as the delivery mechanism.

## 7. Conclusions

Experimental studies support cytoprotective and immunoregulatory effects of IL-15 in the kidney, including tubular epithelial survival, preservation of the epithelial phenotype, and attenuation of profibrotic responses. Podocyte studies demonstrate a functional contribution of STAT5 to autophagic regulation. Separately, IL-15-responsive mitochondrial effects have been demonstrated under diabetic conditions. These findings arise from distinct experimental settings and should not be combined into a single established pathway. The net effects of IL-15 depend on the cellular and inflammatory context, and comparable protection in the intact human kidney has not been established. The relevance of these mechanisms to exercise-associated renal stress remains hypothetical. Exercise-associated increases in circulating IL-15 do not establish its skeletal muscle origin, renal delivery, or functional effects on renal cells. Likewise, evidence of muscle–kidney communication in pathological models does not validate an equivalent physiological exercise-induced axis.

Validation requires distinguishing local renal IL-15 from muscle-derived cytokine, characterizing the molecular forms and modes of presentation of IL-15, and demonstrating cell-specific, IL-15-dependent renal outcomes during exercise. Such studies should distinguish biomarker changes from functional impairment and structural injury and characterize recovery over time. This evidence is needed before IL-15 can be considered an established mechanistic mediator or a therapeutic target for exercise-associated renal stress or injury.

## Figures and Tables

**Table 1:** Representative cellular sources, receptor expression and signaling, and functional effects of the IL-15/IL-15 receptor system in selected tissues.

Cell Type or Compartment	IL-15 Production and Presentation	Receptor Expression, Regulation, and Signaling Evidence	Main Reported Effects	Experimental Models and Interpretive Considerations	Ref.
BLOOD AND LYMPHOID ORGANS					
Dendritic cells, monocytes/ macrophages, and stromal cells	Produce IL-15 and form IL-15/IL-15Rα complexes that support membrane-associated presentation and spatially restricted signaling.	In the studies summarized here, these cells act primarily as IL-15-producing and presenting cells; direct responses to IL-15 may also occur.	Priming of NK cells; maintenance of IFN-γ production; regulation of CD8^+^ memory T-cell generation and homeostasis; maintenance of IL-15-dependent lymphocyte populations.	Activated human monocytes and NK cells; conditional knockout and genetically modified mouse models; reporter models; bone marrow chimeras; flow cytometry; tissue imaging.	[89,90,91,92]
NK cells, CD8^+^ memory T cells	Not generally considered primary physiological sources of IL-15.	Act primarily as signal-recipient cells through CD122/γc following presentation of the IL-15/IL-15Rα complex.	NK cell development, survival, and priming; homeostatic maintenance of CD8^+^ memory T cells. Intense or sustained signaling additionally activates an mTOR-dependent metabolic and cytolytic program in NK cells; prolonged supraphysiological stimulation may be accompanied by metabolic exhaustion.	IL-15- and IL-15Rα-deficient mice; primary murine and human NK cells; flow cytometry; cytotoxicity and metabolic assays.	[4,93,94]
SKELETAL MUSCLE					
Skeletal muscle tissue and myofibers	*IL15* mRNA and IL-15 protein have been detected in skeletal muscle tissue.	Receptor components have been reported, but direct functional responses of mature myofibers to IL-15 in vivo remain incompletely characterized.	IL-15 expression depends in part on muscle fiber-type composition. Resistance exercise may increase *IL15* mRNA without necessarily elevating IL-15 protein levels in muscle tissue or plasma. Experimental enhancement of muscle IL-15 secretion has been shown to affect adipose tissue.	Human muscle biopsies; RT-PCR; immunoblotting; immunohistochemistry; genetically modified mice.	[95,96]
Myoblasts and myotubes	IL-15 and IL-15Rα expression has been detected in myogenic cultures; inflammatory regulation has been reported under specific experimental conditions.	Direct responses to recombinant IL-15 have been demonstrated.	Increased cellular protein content and myosin heavy-chain abundance; reduced proteolysis; enhanced myogenic differentiation and oxidative programming; maintenance of CD8^+^ T-cell responses under inflammatory conditions.	Primary human myogenic cells; murine myotubes and C2C12 cells; overexpression studies; co-cultures with CD8^+^ T cells.	[96,97,98,99]
HEART					
Cardiomyocytes	The primary physiological source of IL-15 in the normal myocardium has not been established.	IL-15Rα, CD122, and γc/CD132 have been detected.	STAT3- and PI3K–ERK1/2-dependent enhancement of cell survival during hypoxia/reoxygenation; in mice, post-infarction IL-15 administration reduced cardiomyocyte death and scar formation and improved cardiac function.	Isolated adult mouse cardiomyocytes; mouse models of myocardial infarction; echocardiography; histology; immunohistochemistry.	[64,86]
CENTRAL NERVOUS SYSTEM					
Astrocytes and microglia	Astrocytic IL-15 production is upregulated during ischemic and hemorrhagic injury; IL-15 and IL-15Rα have been detected in microglia.	Receptor components and JAK-dependent responses have been demonstrated primarily in microglia, whereas the autonomous response of astrocytes remains less well characterized.	Astrocyte-dependent accumulation and activation of NK cells and CD8^+^ T cells during ischemia; enhancement of local astrocyte–microglia interactions during intracerebral hemorrhage.	Primary microglia cultures; RNA and protein analyses; assessment of signaling-protein phosphorylation; mouse models of ischemic and hemorrhagic brain injury.	[78,80,100,101]
Neurons and neural progenitor cells	The significance of these cells as a physiological source of IL-15 remains poorly established.	IL-15Rα expression and functional responses have been demonstrated in neurons, hippocampal slices, and neural progenitor cell cultures.	Modulation of GABAergic and glutamatergic transmission; involvement of the IL-15Rα system in hippocampus-dependent memory; neuronal cytoprotection under ischemic stress; stimulation of neural progenitor-cell proliferation and self-renewal.	Hippocampal slices; patch-clamp recording; single-cell RT-qPCR; mice with systemic IL-15Rα deficiency; primary neuronal cultures; ischemia models; neural stem-cell cultures.	[81,102,103,104]
KIDNEY					
Tubular epithelial cells (TECs)	Endogenous IL-15 production has been reported. The relative contributions of soluble IL-15 and membrane-associated presentation remain incompletely defined.	IL-15Rα, IL-2/IL-15Rβ (CD122), and γc have been detected. JAK/STAT-associated responses and ERK1/2 phosphorylation have been reported in renal epithelial models.	Autocrine support of cell survival and reduced apoptosis during experimental nephritis; preservation of E-cadherin expression; attenuation of extracellular matrix accumulation in experimental renal fibrosis; maintenance of lymphocyte proliferation in mixed cultures.	Primary human TECs/PTECs, UUO mouse models, and IL-15-deficient mice with nephritis. Individual signaling branches were examined in different models and should not be presented as a single established cascade. Findings in cultured human cells do not establish cytoprotection in the intact human kidney.	[6,8,20,21]
Podocytes	Endogenous IL-15 production by podocytes remains poorly characterized. Muscle-derived IL-15 has been implicated in a DKD model.	Responses involving IL-2/IL-15Rβ/γc and JAK/SYK-associated signaling have been reported. IL-15-induced STAT5 phosphorylation has been demonstrated in cultured podocytes; STAT5- and OPA1-associated responses were investigated in distinct experimental settings.	Preservation of autophagic flux, mitochondrial mass, and podocyte integrity in experimental glomerular injury; maintenance of OPA1-dependent mitochondrial fusion and respiratory function in a DKD model.	Cultured podocytes, genetically modified mice, human kidney biopsy analyses, mitochondrial imaging, and respiratory assays. These findings do not establish a unified signaling cascade. Human tissue observations should be distinguished from functional protection demonstrated in preclinical models.	[6,7,22,105]
Renal epithelial–immune interactions and CD8^+^ T-cell-associated effects	TEC-derived IL-15 contributes to lymphocyte responses in mixed cultures. The relevant presentation mode remains incompletely defined. Recombinant IL-15 has also been administered in experimental nephritis.	IL-15-dependent lymphocyte responses have been reported, but receptor-subunit regulation and the cellular mechanisms underlying protective CD8^+^ T-cell-associated effects remain incompletely defined.	Lymphocyte proliferation in TEC–PBMC co-cultures; a protective effect potentially involving CD8^+^ T cells following low-dose recombinant IL-15 administration in one nephritis model.	Human TEC–allogeneic PBMC co-cultures and experimental nephritis. These are distinct experimental settings. Results do not establish a general dose-dependent protective effect or relevance to exercise-associated renal stress.	[106,107]
Renal myofibroblasts and interstitial compartment	Experimental exposure to IL-15 or IL-15/IL-15Rα complexes; the endogenous cellular source was not resolved by these interventions.	Reduced matrix production has been reported in renal myofibroblasts. Receptor-subunit regulation and the relative contributions of direct and indirect signaling remain incompletely defined.	Reduced collagen and fibronectin production; decreased MCP-1 expression, macrophage infiltration, and extracellular-matrix accumulation in experimental fibrosis	Renal myofibroblast experiments and a unilateral ureteral obstruction model. Cell-culture effects on matrix production should be distinguished from whole-kidney outcomes, which may also reflect reduced inflammation and epithelial injury.	[21]

Note: Reported signaling components and functional outcomes were investigated in different experimental systems and should not be interpreted as a single integrated IL-15-dependent cascade. Receptor expression or downstream phosphorylation alone does not establish pathway necessity or a causal cytoprotective effect. Studies using cultured human cells or human tissue samples do not, by themselves, demonstrate protection in the intact human kidney.

**Table 2:** Exercise-associated changes in skeletal muscle *IL15* expression, muscle IL-15 protein content, and circulating IL-15 concentrations.

Exercise Protocol	Reported IL-15-Related Outcome	Ref.
A single bout of heavy resistance exercise	Muscle *IL15* mRNA increased approximately twofold at 24 h, without significant changes in muscle IL-15 protein or plasma IL-15 at the investigated time points.	[95]
30 min of treadmill running	Circulating IL-15 levels increased significantly in untrained healthy young men.	[128]
10 km amateur race	Plasma IL-15 levels increased by approximately 50% immediately after the race and remained elevated 24 h post-race.	[132]
A single bout of high-intensity interval exercise (HIIE) on a cycle ergometer	IL-15 increased immediately after a single HIIE bout assessed before and after a 7-month HIIT program; the program did not significantly alter pre- or post-HIIE concentrations.	[130]
Back-squat exercise	IL-15 levels increased at 24 and 48 h post-exercise.	[133]
Resistance exercise	Serum IL-15 concentrations increased by approximately 5.3-fold immediately post-exercise.	[126]
Eccentric resistance exercise	Serum IL-15 levels increased.	[134]
Static resistance exercise	Plasma IL-15 concentrations increased by approximately 50% in athletes but did not change significantly in untrained individuals.	[131]
12-week resistance training	IL-15 levels increased significantly.	[135]
12 weeks of endurance training	Basal skeletal-muscle IL-15 protein content increased by approximately 40%, whereas muscle *IL15* mRNA abundance and plasma IL-15 concentrations remained unchanged.	[136]
12 weeks of regular supervised endurance exercise	No significant changes in IL-15 levels were observed.	[137]
Marathon running	Circulating IL-15 levels decreased immediately after the race and at 24 and 72 h post-race.	[138]
Strenuous endurance exercise: 35 km trail run with a total elevation gain of 940 m	Serum IL-15 levels increased 2.22-fold.	[129]
Endurance exercise	No significant changes in IL-15 levels were observed immediately after the race or at 24 and 48 h post-race.	[131,139]

Note: Muscle transcript abundance, tissue protein content, and circulating IL-15 concentrations are distinct outcomes. Acute responses to individual exercise bouts should be distinguished from changes in resting concentrations or tissue expression after training. Serum and plasma measurements, exercise protocols, and sampling intervals may differ between studies; reported changes should therefore not be compared directly without considering these factors.

**Table 3:** Evidence and limitations for the proposed exercise-associated skeletal muscle–IL-15–kidney axis.

Proposed Step	Evidence in the Studied Setting	Main Finding	Limitation for the Exercise-Associated Axis	Ref.
Exercise → skeletal muscle IL-15 expression	Muscle expression measurements in human and animal studies.	Increased muscle IL-15 transcript abundance has been reported after selected exercise protocols, with responses varying by protocol and sampling time.	Increased transcript abundance does not establish increased protein synthesis, release into the circulation, or a muscle-derived contribution to circulating IL-15.	[10,35,95]
Exercise → circulating IL-15; contribution of skeletal muscle	Direct measurement of circulating concentrations; generally indirect evidence of tissue origin.	Acute increases in circulating IL-15 have been reported after selected exercise protocols.	Concentration measurements do not identify tissue origin or quantify the contribution of skeletal muscle. Responses vary across protocols, sampling times, and study populations.	[126,128,131]
Circulating IL-15 → renal target-cell exposure during exercise	Exercise-specific renal delivery not established in the studies reviewed.	Circulating IL-15 represents a potential route for endocrine exposure.	The studies reviewed do not demonstrate renal target-cell exposure to biologically active, exercise-associated muscle-derived IL-15 or subsequent receptor engagement.	[45]
Renal IL-15R activation during exercise	Functional cellular responsiveness demonstrated outside the exercise setting; exercise-specific activation not established.	Renal epithelial cells and podocytes respond to exogenous IL-15. Studies have identified receptor component expression and receptor-associated signaling, with the specific evidence differing between cell types and experimental models.	Responses to exogenous IL-15 outside the exercise setting do not establish receptor activation during exercise or a requirement for every receptor subunit in all renal cell responses.	[6,8,105]
IL-15 signaling → protection against exercise-associated renal stress or injury	Functional evidence from separate cell-culture and disease models; extrapolation to exercise.	Separate studies have demonstrated beneficial effects of IL-15 treatment and protective functions of associated signaling components involving epithelial survival, autophagic regulation, mitochondrial morphology and respiration, or attenuation of fibrotic responses.	These findings do not establish protection during exercise or a single causal signaling cascade. Cytokine source, formulation, responding cell population, and effects on renal stress, injury, and recovery require exercise-specific causal testing.	[7,20,22]

Note: Evidence categories describe the relationship between findings obtained in the original experimental setting and their applicability to physical exercise; they do not constitute a formal certainty-of-evidence grading system. Distinct signaling responses and functional outcomes should not be interpreted as components of a single established cascade.

## Data Availability

No new data were created or analyzed in this study. Data sharing is not applicable to this article.
